# Rhinocerebral zygomycosis with pansinusitis in a 14-year-old girl with type 1 diabetes: a case report and review of the literature

**DOI:** 10.1186/1824-7288-39-77

**Published:** 2013-12-10

**Authors:** Annalisa di Coste, Francesco Costantino, Luigi Tarani, Vincenzo Savastano, Claudio Di Biasi, Laura Schiavi, Ilaria Ernesti, Taulant Melengu, Marzia Duse

**Affiliations:** 1 Department of Pediatrics, Policlinico Umberto I, “Sapienza” University of Rome, Viale Regina Elena 324, Rome 00161, Italy; 2 Department of Radiology, Policlinico Umberto I, “Sapienza” University of Rome Viale Regina Elena 324, Rome 00161, Italy

**Keywords:** Rhinocerebral zygomycosis, Diabetes, Maxillary sinus, Endoscopic Sinus Surgery, Posaconazole

## Abstract

**Background:**

Zygomycosis is a rare life-threatening fungal infection affecting mostly patients with predisposing conditions such as diabetes mellitus, immunodeficiency, haemochromatosis or major trauma.

**Methods:**

We describe a case of rhinocerebral zygomycosis in a girl with type 1 diabetes and review previous published cases and treatment options.

**Results:**

A 14-year-old girl with type 1 diabetes mellitus occurred with dental pain, facial swelling, ecchymosis and left eye decreased visual acuity, unresponsive to antibiotic therapy. The coltures of the sinusal mucosa were positive for fungal species belonging to the Zygomycetes. She performed antifungal therapy with posaconazole (POS) with a very slow improvement and a poor glycemic control, leading to blindness of the left eye.

**Conclusion:**

Our report adds further awareness on rhinocerebral zygomycosis and emphasizes on urgent diagnosis and timely management of this potentially fatal fungal infection through an adequate treatment.

## Background

Zygomycosis is a rare life-threatening opportunistic fungal infection in humans, that often complicates diabetes mellitus and primary and acquired immunodeficiencies characterised by defects of the cell-mediated immunity. Other predisposing factors include steroid therapy, organ transplantation and cytotoxic chemotherapy [1]. The causative organism is an aerobic saprophytic fungus belonging to the order of *Mucorales* of the class *Zygomycetes*. It is ubiquitous insoil, grows rapidly and constantly discharges spores into the environment [2]. Infection is usually caused by inhalation of sporangiospores or via direct contamination of skin wounds, especially burns. The lungs, nasal cavity and paranasal sinuses, gut and cutaneous tissues are therefore the most common sites for primary infection. At onset, Rhinocerebral Zygomycosis presents with different clinical features such as blindness, cranial nerve palsies, eye proptosis and pain. Fungal hyphae preferentially invade the walls of blood vessels producing thrombi and infarction. The resulting progressive tissue ischaemia and necrosis of deep tissues may include muscle and fat, ultimately leading to multiorgan failure and sepsis. The central nervous system can be invaded by Zygomycetes either contiguously from adjacent paranasal sinuses (rhinocerebral zygomycosis), or haematogenously from a remote site of infection [3]. We report a case of necrotizing invasive rhinocerebral zygomycosis in a 14-year-old girl with type 1 diabetes and review previous reported cases from 1980 to 2012.

## Materials and methods

### Case presentation

**Figure 1:**
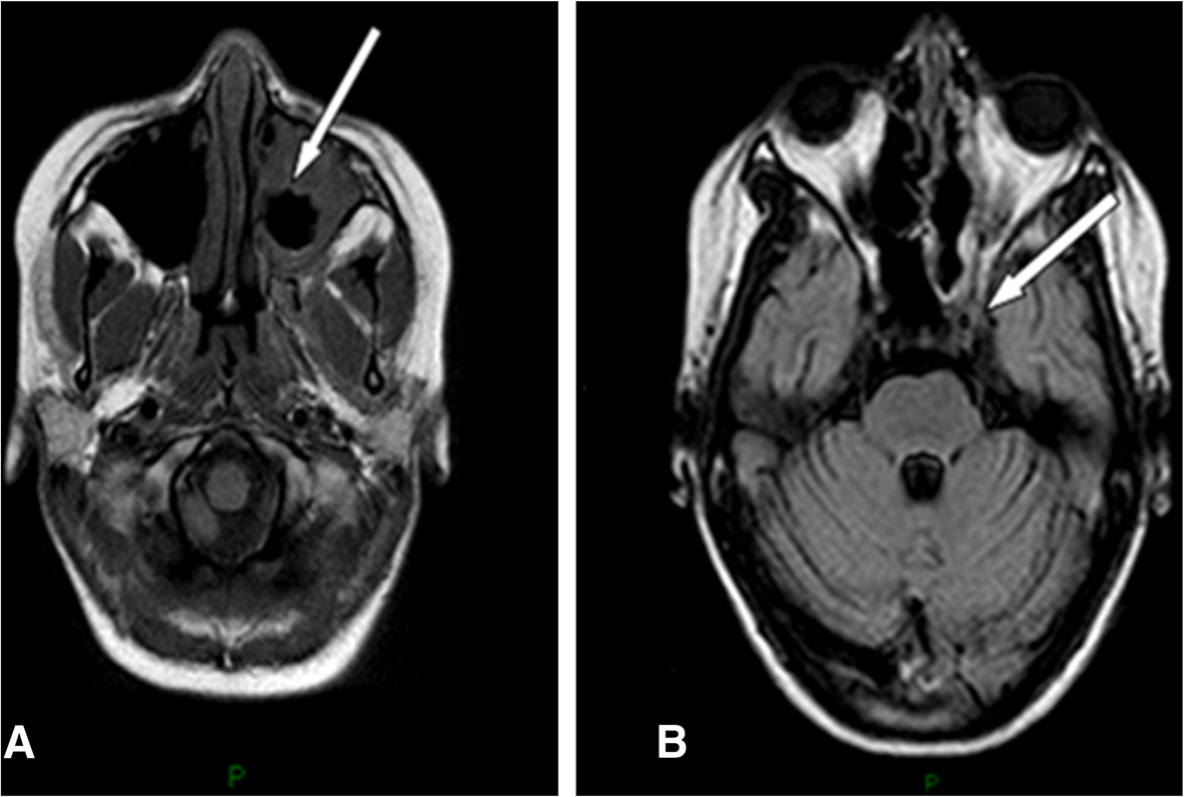
**Magnetic resonance imaging of head. A.** Postgadolinium T1-weighted axial magnetic resonance image showing nonenhancing tissue (arrow) in the left nasal cavity and periorbital site compatible with necrotic mucosa. **B.** Similar technique showing note semiocclusion of the left internal carotid artery (arrow) and thrombosis of the right cavernous sinus, although there was no radiographic evidence of cerebral infarction.

A 14-year-old caucasian girl is followed at our Department of Pediatrics for type 1 diabetes mellitus since the age of three years with poor glycemic control despite regular insulin therapy. She occurred with a 7-day history of dental pain, facial swelling (extending superiorly from the supraorbital margin and inferiorly to the angle of the mouth), and ecchymosis in the left periorbital region with decreased visual acuity and colour vision, unresponsive to antibiotic therapy with amoxicillin. Physical examination showed left-sided facial numbness, lagophthalmos with inability to complete closure of the left eyelid and tongue deviation to the left. Intraorally there were carious lesions and low sensitivity of the upper teeth. Biochemical investigations revealed neutrophilia and increasing of inflammatory markers. The electromyography showed a severe VII nerve damage. Radiographic examination showed haziness of left maxillary sinus with erosion of lateral sinus wall. Magnetic resonance imaging of her head revealed a marked mucosal thickening of the left maxillary sinus extended to the sphenoid, ethmoid and frontal sinus with moderate inflammatory effusion (Figure 1). For three times inflammatory tissue was excised from the sinuses through an Endoscopic Sinus Surgery for microbial eradication and histopathologic examination. The tissue biopsy showed fragments of mucosa lined by respiratory epithelium with chronic aspecific inflammatory infiltrate and growed fungal species belonging to the Zygomycetes. After a 20-day treatment with imipenem, teicoplanin, metronidazole and aciclovir, she was given intravenous amphotericin B to which posaconazole (POS) was immediately replaced, due to the onset of side effects such as hyperglycemia, marked hypothermia and profuse sweating. The follow-up magnetic resonance images showed progression of the disease with significant intracranial extension. The magnetic resonance angiography showed the involvement of the neurovascular retromandibular axis and left cavernous sinus with occlusion of ipsilateral cavernous carotid and increased signal of the walls of the left middle cerebral artery caused by arteritis. She continued antifungal drug therapy and clinic follow-up showing a very slow improvement, a poor glycemic control and many recurrences, leading to blindness of the left eye.

## Results

Zygomycosis is rare in pediatric patients, and there are few reports in the literature. We performed a MEDLINE search for articles published in the English-language literature, ages: 0–18 years, from January 1980 to September 2013. The search terms used were: rhinocerebral zygomycosis or mucormycosis and case report.

Our search yielded a total of 25 articles including 28 case reports (Table 1). Cases included were those with acute zygomycosis infection in the rhinocerebral region diagnosed by histology with or without a positive culture, with hematologic malignancies or type 1 diabetes as predisposing factors. Fifteen out of 28 cases (53.6%) of rhinocerebral zygomycosis from these case series were found in patients with type 1 diabetes, twelve (42.8%) in patients with hematologic malignancies and one (3.6%) in a patient with autoimmune hepatitis.

**Table 1:** Pediatric rhinocerebral zygomycosis: review of the literature from 1980 to 2013

**Author**	**Cases (Number)**	**Sex**	**Ages (years)**	**Underlying condition**	**Treatment**	**Outcome**
Kilpatrick et al. [4]. 1984	1	F	18	DM 1	Surgery, antifungal therapy	Survived with left orbital exenteration and ethmoidectomy.
Kemper et al. [5]. 1993	1	F	18	DM 1	Surgery, L-AmB, HOT	Survived with right eye exenteration
Garces et al. [6]. 1994	1	M	6	Hematologic malignancy	Surgery	Deceased
Moll et al. [7]. 1994	1	Unknown	12	DM 1	Surgery, antifungal therapy	Survived with minimal left cranial nerve palsies
Shah et al. [8]. 1997	1	M	15	DM 1	L-AmB and HOT	Survived with a ventriculoperitoneal shunt, right nonreactive pupil, minimal residual physical disability
Kameh et al. [9] 1997	1	M	13 months	DM 1	Surgery, L-AmB	Deceased
Adler et al. [10]. 1998	1	M	16	DM 1	Surgery, L-AmB	Survived
Lee et al. [11] 1998	1	M	10	DM 1	Surgery, L-AmB	Survived with open craniotomy for abscess resection
Khanna et al. [12]. 1998	1	F	11	DM 1	Surgery, L-AmB	Deceased
Parkin et al. [13]. 2000	2	M; M	13; 3	Hematologic malignancy	G-CSF, L-AmB	Deceased for other cause; survived after bone marrow transplant
Ryan et al. [14]. 2001	2	F; M	9;3	Hematologic malignancy	Surgery, L-AmB	Deceased
Gessesse et al. [15]. 2001	1	M	11	DM 1	Unknown	Deceased
Wehl et al. [16]. 2002	1	M	9 months	Hematologic malignancy	L-AmB	Deceased
Hamilton et al. [17]. 2003	1	M	14	DM 1	Surgery, antibiotics, HOT	Survived with enucleation of right eye
Simmons et al. [18]. 2005	1	F	8	DM 1	Surgery, L-AmB, HOT, Interferon-G, POS, GM-CSF	Survived
Sorensen et al. [19]. 2006	1	F	10	Hematologic malignancy	L-AmB, POS, Caspofungin, surgery	Deceased for other cause
Popa et al. [20] 2008	1	F	7	Hematologic malignancy	L-AmB	Survived after neurosurgical debridement of the left temporal abscess
Garner et al. [21]. 2008	2	M; M	5; 15	Hematologic malignancy	L-AmB, POS	Survived
Ganesh et al. [22] 2008	1	M	14	DM 1	L-AmB, itraconazole	Survived
Tarani et al. [23]. 2009	1	F	12	DM 1	L-AmB, GM-CSF, HOT, surgery, POS	Survived
Ibrahim et al. [24]. 2009	1	F	12	DM 1	L-AmB, POS	Survived
Skiada et al. [3]. 2009	1	M	2	Hematologic malignancy	L-Am B, POS	Vegetative state
Safder et al. [25]. 2010	1	F	12	Autoimmune hepatitis	Surgery, L-AmB	Survived with right eye exenteration
Nirmala et al. [26]. 2011	1	M	12	DM 1	L-AmB	Survived
Prasad et al. [27]. 2012	1	F	15	Hematologic malignancy	Surgery and L-AmB	Deceased

Abbreviations: *M* Male, *F* Female, *DM 1* Type 1 Diabetes, *L-Amb* Liposomal Amphotericin B, *HOT* Hyperbaric Oxygen Therapy, *POS* Posaconazole, *GM-CFS* Granulocyte Macrophage Colony Stimulating Factor.

In described pediatric cases rhinocerebral zygomycosis presents with a characteristic onset: cranial nerve palsies (66.7% of cases), facial/eye swelling or blindness (40%), eye proptosis (33.3%), periorbital cellulitis or pain (20%), epistaxis, headaches, nasal discharge, decreasing consciousness, dysarthria and otalgia (6.7%).

All cases have the involvement of at least one eye. All patients underwent surgery and/or therapy with amphotericin B and other antifungal agents. The 25% of patients with type 1 diabetes has passed the infection uncomplicated, while the 40% underwent to complications such as eye exenteration, partial loss of vision, invasive surgery and nerve palsies. The overall mortality was 20% in cases with type 1 diabetes and 50% in cases with other predisposing factors. The mortality rate was significantly higher when the central nervous system was involved compared to sinus or sino-orbital involvement only.

## Discussion

The Zigomycosis’ predisposing factors are uncontrolled diabetes, lymphomas, leukemias, renal failure, organ transplant, long-term corticosteroid and immunosuppressive therapy [28]. Hyperglycemia, usually with an associated metabolic acidosis, is responsible for impaired neutrophil function, neuropathies and vascular insufficiency leading to a diminished resistance to infections and altered tissue response. In ketoacidosis, the acid environment due to the increase in glucose levels and the increase in levels of free iron ions favour fungal growth [2].

The clinical onset of the Rhinocerebral Zygomycosis in our report and in most cases is characterized by cranial nerve palsies, facial/eye swelling and blindness, less frequently by decreasing consciousness.

Mucormycosis is known for having a very poor prognosis: survival rates are currently thought to exceed 80%. Even with successful treatment, Mucorales can reappear during future courses of chemotherapy and neutropenia [29]. There may be predictive factors on the evolution of complications: patients who presented with “blindness” seem to have a higher prevalence of survival (p ≤ 0.04) while patients who presented with “decreased consciousness” seem to have an higher prevalence of death (p ≤ 0.04). With the advent of potent antifungal medications, a combination of surgery, medication and correction of the underlying conditions has provided better outcomes [26]. Surgery needs to be radical with an aim to remove all devitalized tissue, and has to be repeated based on disease progression. The new triazole POS has a broad antifungal spectrum against filamentous fungi. The use of POS seems to be associated with a high prevalence of survival with or without outcome (p ≤ 0.04). This is important as standard therapy with amphotericin B often fails [23].

## Conclusion

The zygomycosis may present with specific clinical symptoms in patients with predisposing conditions. “Blindness” seems to be correlated with survival predictive factors while “decreased consciousness” seems to be associated with severe outcome.

Early diagnosis of zygomycosis and meticulous broad spectrum antifungal therapy are necessary to avoid the further spread of infection, which may lead to high morbidity and mortality. The timely use of POS alone or in combination with other therapies seems to be associated with a high prevalence of survival with or without outcome.

Larger studies and populations are needed to test if these relationships are casual or real. It is important to understand and implement the treatment options which can help to manage these patients with peculiar onset before the coltures results. Knowledge of potentially devastating complications can help to prevent the unfortunate consequences.

Written informed consent was obtained from the patient’s parents for publication of this Case report and any accompanying images. A copy of the written consent is available for review by the Editor-in-Chief of this journal.

## Competing interest

The authors declare no conflict of interest with any financial organization regarding the material discussed in the manuscript. Authors do not have sources of funding.

## Authors’ contribution

AdC, FC, LT, VS, CDB, LS, IE, TM, and MD participated in the sequence alignment and drafted the manuscript. All authors contributed equally to the manuscript. All authors read and approved the final manuscript.
